# Neoplastic lesions in domestic pigs detected at slaughter: literature review and a 20-year review (1998–2018) of carcass inspection in Catalonia

**DOI:** 10.1186/s40813-021-00207-0

**Published:** 2021-04-07

**Authors:** Antonia Morey-Matamalas, Enric Vidal, Jorge Martínez, Jaume Alomar, Antonio Ramis, Alberto Marco, Mariano Domingo, Joaquim Segalés

**Affiliations:** 1 Servei de Diagnòstic de Patologia Veterinària (SDPV), Departament de Sanitat i d’Anatomia Animals, Universitat Autonòma de Barcelona, 08193 Bellaterra (Cerdanyola del Vallès), Barcelona, Spain; 2 IRTA, Centre de Recerca en Sanitat Animal (CReSA, IRTA-UAB), Campus de la Universitat Autònoma de Barcelona, 08193 Bellaterra (Cerdanyola del Vallès), Barcelona Spain

**Keywords:** Tumor, Neoplasm, Slaughterhouse, *Sus scrofa*, Swine, Food inspection, Surveillance, Lymphoma, Retrospective study

## Abstract

**Background:**

The present paper reviews the occurrence of neoplasms in swine and presents a case series of 56 tumors submitted to the Slaughterhouse Support Network (*Servei de Suport a Escorxadors* [SESC] IRTA-CReSA]) from slaughtered pigs from 1998 to 2018 (April) in Catalonia (Spain). The aim of the study was to describe the spectrum of spontaneous neoplastic lesions found in slaughtered pigs and to compare the reported tumor cases with previous published data. Lymphoid neoplasms were characterized and classified using the WHO classification adapted for animals.

**Results:**

The most reported neoplasm during this period was lymphoma (28). Within lymphomas, the B-cell type was the most common, being the diffuse large B-cell lymphoma (15/28) the most represented subtype. Other submitted non-lymphoid neoplasms included melanoma (7), nephroblastoma (3), mast cell tumor (2), liposarcoma (2), osteochondromatosis (2), papillary cystadenocarcinoma (1), peripheral nerve sheath tumor (1), lymphoid leukemia (1), fibropapilloma (1), hemangiosarcoma (1), hepatoma (1), histiocytic sarcoma (1), pheochromocytoma (1) and osteosarcoma (1).

**Conclusions:**

The existence of a well-established Slaughterhouse Support Network allowed the compilation of comprehensive data for further epidemiological and pathological studies, particularly about less commonly reported lesions in livestock such as neoplasms in pigs.

**Supplementary Information:**

The online version contains supplementary material available at 10.1186/s40813-021-00207-0.

## Background

Spontaneous neoplasms in pigs are infrequent because most animals are slaughtered before reaching maturity. In this species, neoplasia does not frequently cause clinical signs, thus it is typically diagnosed as an incidental finding in carcasses at slaughterhouse [1]. Detection during meat inspection is based on visual inspection and time and workload restrictions do not allow for detailed investigation and macroscopic descriptions. In addition, the recent risk-based European Commission Regulation (EU) 2019/627 of 15 March 2019 only allows visual inspections on pig carcasses and viscera, hence certain diseases can go undetected [2] . Moreover, macroscopic lesions are usually not distinct or specific and some neoplastic and infectious disease lesions have similar appearance (e.g. fungal or mycobacterial granulomas); therefore, most neoplasms are undetected or misdiagnosed without histological examination.

In swine, the most commonly reported neoplasms are those of lymphoid origin [3–13] and, to a lesser extent, melanomas [14–16].

Several abattoir surveys reviewed the different neoplasms found in slaughtered domestic animals and their incidence in the past 50 years. In the late 1960s, a large histologic survey of neoplasms in slaughtered animals was carried out throughout 100 abattoirs in Great Britain, which led to a comprehensive description of neoplastic lesions in swine, bovine and sheep including the type of neoplasm and incidence encountered per organ [3, 17–23]. Previously, in 1963, Brandly and Migaki presented a 8-year survey which described different neoplasms detected in domestic animals slaughtered in the United States [24]. Other surveys conducted in Denver (United States) [25], Canada [26], Czech Republic [27] and the Republic of South Africa [28] were also published during that period. Since then, only sporadic case reports and occasional case study series [29] have been published. A table summarizing the collected reported neoplasms in slaughter pigs from the 1960’s until the present is presented in the Additional file 1.

The increase in miniature pigs – Vietnamese and Pot-bellied pigs - as pets prompted a study of neoplastic conditions due to its longer lifespan. A retrospective study in 63 pot-bellied pigs summarized the incidence of different neoplasms in these breeds [30]. Frequently identified neoplasms were those affecting the reproductive tract (including leiomyoma and leiomyosarcoma), liver (hepatocellular and biliary carcinomas) and gastrointestinal tissues (colonic carcinoma) [30].

As mentioned earlier, lymphoma is the most common neoplasm in pigs. All ages and both sexes are affected, with a higher incidence reported in females. The most common subtype is the multicentric form, characterized by enlargement of visceral lymph nodes (rather than peripheral) with or without infiltration of internal organs, being the liver, spleen and kidneys the most commonly affected. The thymic form is common in young piglets (< 3 weeks) [31]. Regarding the histological classification of these neoplasms, no published data using the WHO classification of lymphoid neoplasms has been performed to a large series of porcine lymphomas [31].

In the present paper, neoplasms detected in 56 slaughtered pigs in Catalonia from 1998 to April 2018 are described. The aim of the study was to describe the spectrum of spontaneous neoplastic lesions found in pigs slaughtered and to compare the reported neoplasms in the present case series with previously published data. Additionally, immunohistochemical characterization and classification of lymphoid neoplasms following the WHO guidelines was applied.

## Results

### Literature review

To the authors’ knowledge, based on the present review of the literature, 799 cases of spontaneous neoplasms were reported in 798 pigs dating between 1956 and January 2021 in a total of 65 selected papers. A summary of cases of spontaneous neoplasia in pigs arranged by reference in chronological order and including detailed case information (age, sex, numbers of animals affected, breed, affected organs/tissue, metastases, tumor type, and type of report for each case in which such information were available) is provided in Additional file 1.

Three categories of publication have been established: (1) abattoir surveys, (2) case series studies and (3) single case reports. Of the 65 selected papers, 12 (18.5%) were abattoir surveys from Great Britain [3, 17–23], United States [24], Canada [26], Czech Republic [27], and South Africa [28], which included a total of 329 (41.2%) out of 799 neoplasms. In abattoir surveys, the most reported neoplasm was lymphoma (185/329, 56.2%), followed by nephroblastoma (65/329, 19.8%), hepatoma (7/329, 2.1%), fibrosarcoma (6/329, 1.8%), hemangioma (6/329, 1.8%) and squamous cell carcinoma (SCC) (6/329, 1.8%). A list of the type of reported neoplasms, organ affected (primary) and frequency is shown in Table 1.

**Table 1:** Literature review, abattoir / meat inspection surveys

Type of tumor	Organ affected (primary)	No.
Adenocarcinoma	Gallbladder	1
Adenomyoma	Uterus	1
Adnexal adenoma	Skin	1
Adrenal adenoma	Adrenal gland	1
Adrenocortical carcinoma	Adrenal gland	1
Bronchogenic carcinoma	Lung	1
Carcinoma, undifferentiated	NA	4
Carcinoma, hepatocellular	Liver	3
Chondrosarcoma	Lung	1
Fibroma	Kidney (capsule) (1), peritoneum and pleura (1)	2
Fibrosarcoma	Sublumbar connective tissue (1), skin (1), skeletal muscle (1), lung (1), spleen (1), kidney (1)	6
Giant cell carcinoma	Various organs	1
Granulosa cell tumor	Ovary	3
Hemangioendothelioma	Liver	2
Hemangioma	Skin (pinna) (1), gastrointestinal tract (1), peritoneum and pleura (2), spleen (2)	6
Hemangiosarcoma	Peritoneum and pleura	1
Hepatoma	Liver	7
Hypernephroma	Kidney	1
Islet-cell tumor	Pancreas	1
Leiomyoma	Ovary (2), genital tract (1)	3
Leiomyosarcoma	Uterus	2
Lymphoma		185
Multicentric lymphoma	Various organs	150
Thymic lymphoma	Thymus	35
Melanoma		5
Benign (melanocytoma)	Skin	4
Malignant	Skin	1
Mesothelioma	Peritoneum and pleura	3
Multiple myeloma	Hematopoietic	1
NA	NA	4
Nephroblastoma		64
Benign	Kidney	63
Malignant	Kidney	1
Papillary serous cystadenocarcinoma	Ovary	2
Peripheral Nerve Sheath Tumor		3
Neurofibroma-neurilemmoma	Nervous tissue	3
Pheochromocytoma	Adrenal gland	1
Renal carcinoma	Kidney	2
Reticulum cell sarcoma	Lymph nodes	1
Squamous cell carcinoma	Skin (5), orbit skin (1)	6
Thymoma	Thymus	1
Transitional (urothelial) cell carcinoma with squamous metaplasia	Kidney, renal pelvis	1
Wart	Skin	1
TOTAL		329

List of reported neoplasm types (alphabetic order), organ affected (primary) and number. The number of cases per organ is indicated in brackets when a neoplasm has been described in more than one organ. Metastases to other organs are not included

*NA* Not available

Thirteen [6, 8, 10, 11, 15, 16, 29, 32–37] (20.0%) out of the 65 selected papers were case series studies of different types of neoplasm, representing 429 (53.7%) out of 799 neoplasms. In this subgroup of cases, the most reported neoplasm was melanoma (214/429, 49.9%), followed by nephroblastoma (81/429, 18.9%), lymphoma (75/429, 17.5%), hemangioma (21/429, 4.9%), rhabdomyoma (10/429, 2.3%) and leiomyoma (8/429, 1.9%). A list of the type of reported neoplasms, organ affected (primary) and frequency is shown in Table 2.

**Table 2:** Literature review, case series / study papers

Type of tumor	Organ affected (primary)	No.
Ameloblastoma	Oral cavity	1
Chrondroma	Trachea	1
Cyst-adenoma	Uterus	1
Glioblastoma	Cerebrum	1
Granulocytic sarcoma	Various organs	1
Fibroma	Uterus (3), cervix/vagina (1)	4
Hemangioma	Ovarian (18), cutaneous (2), meningeal (1)	21
Hemangiosarcoma	Testicular (1), cutaneous (1)	2
Leiomyoma	Uterus (7), pylorus (1)	8
Lipoma	Mesentery	1
Lymphoma		75
Multicentric lymphoma	Various organs	64
Ileal lymphoma	Ileum	11
Melanoma		214
Regressing	Skin	174
Benign (melanocytoma)	Skin	37
Malignant	Skin	3
Nephroblastoma		81
Benign	Kidney	79
Malignant	Kidney	2
Papilloma	Penile, oral, cutaneous	3
Rhabdomyoma, congenital	Heart	10
Rhabdomyosarcoma	Skin	5
TOTAL		429

List of reported neoplasm types (alphabetic order), organ affected (primary) and number. The number of cases per organ is indicated in brackets when a neoplasm has been described in more than one organ. Metastases to other organs are not included

The last group of papers included single case reports, with a total of 40 (61.5%) papers collected [9, 12–14, 38–73], representing a total of 41 neoplasms in 40 pigs. Within this group, peripheral nerve sheath tumors (PNST) (4/40, 10.0%), lymphoma (3/40, 7.5%), mast cell tumor (2/40, 5.0%), hepatocholangioadenoma (2/40, 5.0%), fibropapillomatosis (2/40, 5.0%), osteosarcoma (2/40, 5.0%), rhabdomyoma (2/40, 5.0%) and carcinoma (2/40, 5.0%) were the most reported ones. A list of the type of reported neoplasms, organ affected (primary) and frequency is shown in Table 3.

**Table 3:** Literature review, case reports

Type of tumor	Organ affected (primary)	No.
Carcinoma	Mammary gland (1), uterus (1)	2
Carcinosarcoma, endometrial	Uterus	1
Eosinophilic granulocytic sarcoma	Bone	1
Fibropapillomatosis	Skin	2
Fibrosarcoma	Subcutaneous tissue	1
Ganglioneuroma	Small intestine	1
Hamartoblastoma	Spleen	1
Hamartoma, fibroepithelial	Skin	1
Hemangiosarcoma	Meninges (brain)	1
Hepatocholangioadenoma	Liver	2
Histiocytoma, fibrous, malignant	Spleen	1
Histiocytosis, congenital	Skin	1
Leydig cell tumor	Testis	1
Lipoma, ossifying	Intracranial	1
Liposarcoma	Perirenal fat tissue	1
Luteoma, malignant	Ovary	1
Lymphoma		3
Signet ring cell	Lymph node and liver	1
Multicentric, T-cell rich / B-cell rich	Lymph node	1
Multicentric, T-cell	Various organs	1
Mast cell tumor	Skin	2
Melanoma, malignant	Skin	1
Myeloid leukemia	Bone marrow	1
Myofibroblastic sarcoma	Diaphragm	1
Osteochondromatosis	Bone	1
Osteoma	Bone (oral cavity)	1
Osteosarcoma	Bone (hard palate (1), mandible (1))	2
Peripheral Nerve Sheath Tumor		4
Cutaneous pigmented neurofibroma	Skin	1
Cutaneous plexiform schwannoma	Skin	1
Malignant	Lung (1), thoracic limb (1)	2
Pheochromocytoma, malignant	Adrenal gland	1
Rhabdomyoma, congenital	Heart	2
Sertoli, malignant	Testis	1
Squamous cell carcinoma	Skin	1
Teratoma	Cerebellum	1
TOTAL		41

List of reported neoplasm types (alphabetic order), organ affected (primary) and number. The number of cases per organ is indicated in brackets when a neoplasm has been described in more than one organ. Metastases to other organs are not included

### Neoplastic lesions in swine

A collection of 56 neoplastic lesions was compiled retrospectively between January 1998 and April 2018. Since the implementation of the SESC in 2008 as slaughterhouse support service, the number of detected neoplastic cases increased remarkably.

One organ was submitted in 12 out of 56 cases (21.4%), while multiple organs were submitted in 44 out of 56 (78.6%). The most frequently submitted organs were lymph nodes (22/56, 39.3%), liver (21/56, 37.5%) and kidney (20/56, 35.7%), followed by spleen (8/56, 14.3%), skin (7/56, 12.5%), skeletal muscle (7/56, 12.5%) and bone (6/56, 10.7%).

In the present case series, 33 (58.9%) submissions were from fattening pigs (6-month to 1-year-old) and 23 (41.1%) from adults (sow or boar). In regards of sex distribution, 27 (48.2%) were females, 19 (33.9%) were males, and in 10 (17.9%) cases the gender was not available. Out of the 27 females, 18 (66.6%) were adult while only 5 (26.3%) out of the 19 males were adults. The females were mainly breeding sows, which were slaughtered at high parity numbers. Within the group of fattening pigs, animals commonly reached the slaughterhouse at 6 months of age, and less commonly, slightly later.

**Fig. 1:**
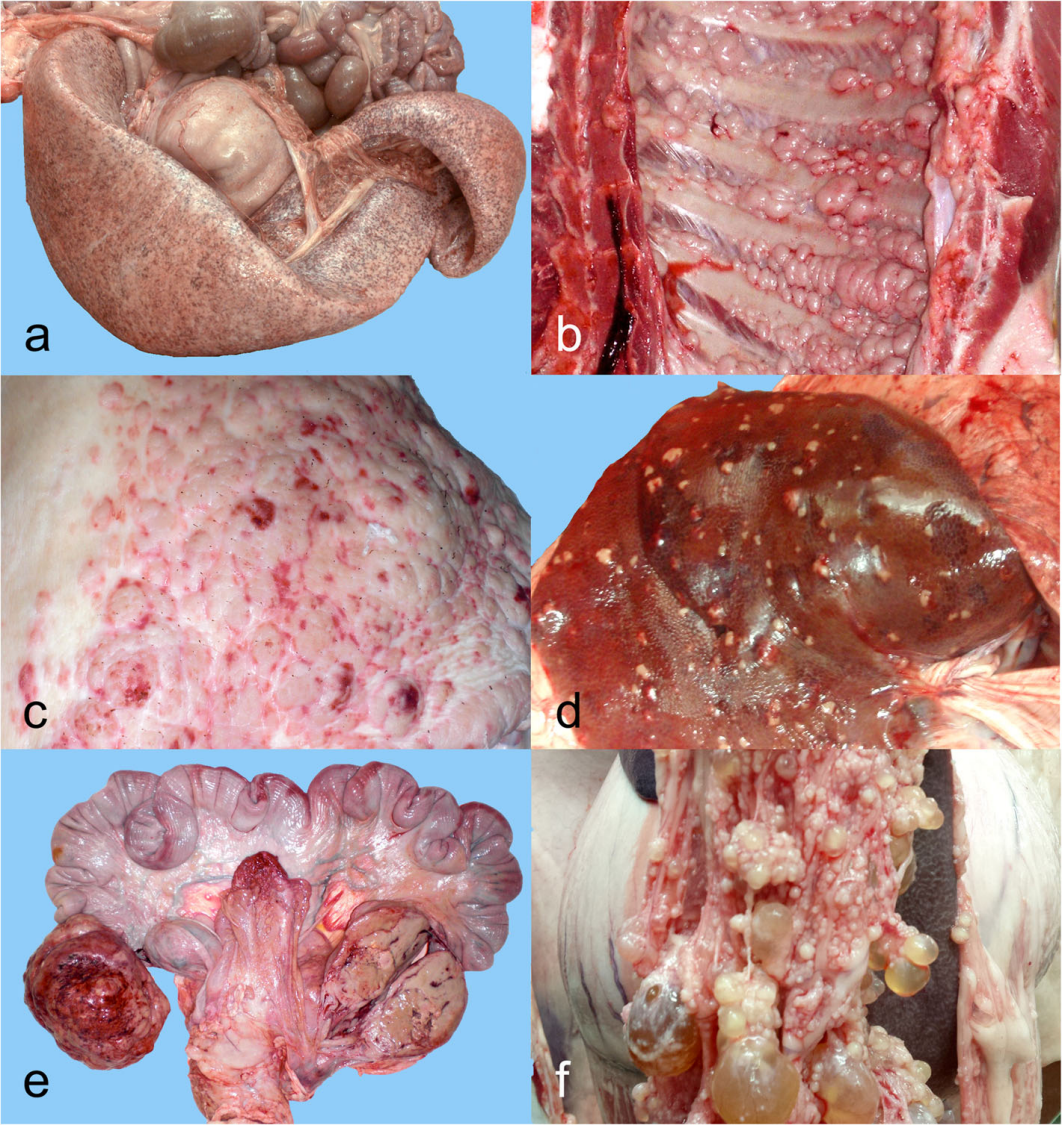
Neoplastic lesions in pig carcasses. **a** Mast cell tumor, spleen, 6 month-old pig. Case 41. Diffuse enlargement and paleness of the spleen. **b** Undifferentiated sarcoma, thoracic pleura, 3 year-old sow. Case 14. Multifocal, coalescing, whitish nodular lesions throughout the parietal pleura. **c** Multicentric lymphoma, hindlimb skin (scalded), 3 year-old sow. Case 33. Multifocal-to-coalescing raised lesions on the skin. **d** Multicentric lymphoma, liver, fattening pig (6–8 months old). Case 17. Multifocal generalized white nodular lesions protruding over the liver capsule. **e** Multicentric lymphoma, ovaries, 2.5 year-old sow. Case 23. Bilateral ovaric nodular enlargement, hemorrhagic on the surface and whitish when sectioned. **f** Papillary cystadenocarcinoma, abdominal serosa, 3 year-old sow. Case 43. Multiple clustered (grape –like) fluid filled cystic lesions of variable diameters throughout the abdominal serosa

Regarding the type of neoplasia among the 56 cases, 28 (50%) were classified as lymphoma, 7 (12.5%) were melanomas, 3 (5.4%) nephroblastomas, 2 (3.6%) mast cell tumors, 2 (3.6%) liposarcomas and 2 (3.6%) osteochondromatosis. Other neoplastic lesions recorded in this series, with only 1 (1.8%) representative each were: leukemia, fibropapilloma, hemangiosarcoma, hepatoma, histiocytic sarcoma, pheochromocytoma, osteosarcoma, papillary cystadenocarcinoma, peripheral nerve sheath tumor and 3 unclassified neoplasms (including an undifferentiated sarcoma, a malignant round/polygonal cell (most likely mesothelioma) and a non-classified malignant round cell tumor) (Fig. 1). The proportion of all neoplasms is shown in Table 4.

**Table 4:** Types of neoplasia detected in slaughtered pigs in Catalonia

Types of neoplasia detected	Number	%
Lymphoma	28	50,0
Melanoma, malignant	7	12,5
Nephroblastoma	3	5,4
Unclassified	3	5,4
Osteochondromatosis	2	3,6
Liposarcoma	2	3,6
Mast cell tumor	2	3,6
Histiocytic Sarcoma	1	1,8
Hemangiosarcoma	1	1,8
Fibropapilloma	1	1,8
Pheochromocytoma	1	1,8
Osteosarcoma	1	1,8
Leukemia	1	1,8
Hepatoma	1	1,8
Peripheral Nerve Sheath Tumor	1	1,8
Papillary Cystadenocarcinoma	1	1,8
TOTAL	56	100,0

Number and percentage (%) of cases per each type of neoplasia detected in slaughtered pigs in Catalonia between January 1998 to April 2018

Information on all studied cases is summarized in Additional file 2 including information about age, breed, sex, organ, gross findings, meat inspector suspicion and final diagnosis.

Examined together, the cases of neoplasia in pigs found in the literature and the ones described in this retrospective study add up to a total of 855 neoplasms in 854 pigs, with 291 (34%) being lymphomas.

### Classification of lymphoid neoplasms in pigs

In the present case series, most of the lymphoid neoplasms (24/29, 82.8%) were multicentric in distribution, three were unclassified and one was a thymic lymphoma (see Additional file 2). A single case of B-cell leukemia was recorded in a male fattening pig. Of the 29 pigs with lymphoid neoplasms, 16 (55.2%) were females, 10 (34.5%) males and in three cases (10.3%) gender was not indicated. Regarding the age, 14 (48.3%) out of 29 were adults (10 [71.4%] females and four [28.6%] males), 14 (48.3%) were fattening pigs (six [42.8%] females and five [35.7%] males), and from three cases (21.5%) the age was not available.

**Fig. 2:**
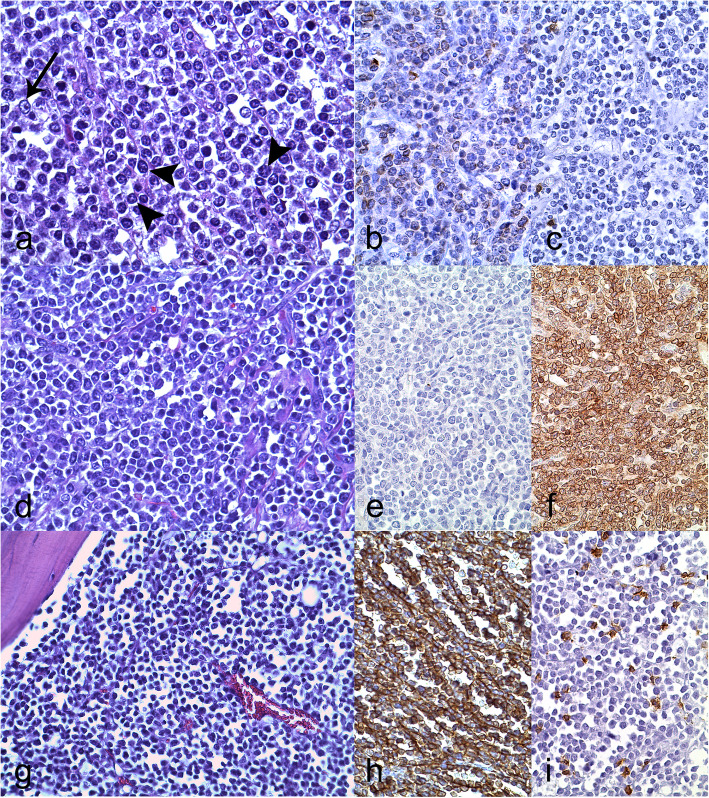
Lymphoma classification in pigs. Representative images of different lymphoma subtypes. **a-c** Diffuse large B-cell lymphoma – centroblastic (DLBCL-CB), spleen, adult sow, case 20. **a** Splenic nodule composed of dense sheets of lymphoid cells arranged in a diffuse pattern, with intermediate to large nuclear size and scant cytoplasm, cells often exhibit multiple nucleoli (centroblastic) (arrowheads), rarely immunoblastic cells (one single central nucleoli) (black arrow) are observed. HE, 630x. **b** Most infiltrating cells have a mild to moderate cytoplasmic CD20 immunolabelling. IHC for CD20, hematoxylin counterstain, 400x. **c** Infiltrating cells are negative to CD3 immunolabeling, very few small mature, non-neoplastic lymphocytes show positive CD3 immunolabeling. IHC for CD3, hematoxylin counterstain, 400x. **d-f** Unspecified, peripheral T-cell lymphoma (PTCL), lymph node, adult sow, case 21. **d** Effacing the normal lymph node architecture there are dense sheets and rows of lymphoid cells arranged in a diffuse pattern, with intermediate to large nuclear size and scant. HE, 630x. **e** Infiltrating cells are negative to CD20 immunolabeling. IHC for CD20, hematoxylin counterstain, 400x. **f** Most infiltrating cells have moderate to strong cytoplasmic CD3 immunolabelling. IHC for CD3, hematoxylin counterstain, 400x. **g-i** B-lymphoblastic leukemia/lymphoma (B-LBL), vertebra, bone marrow, fattening pig, case 47. **g** Dense sheets and rows of lymphoid cells arranged in a diffuse pattern, with intermediate to large nuclear size and scant cytoplasm infiltrating the bone marrow. HE, 400x. **h** Most infiltrating cells have moderate to strong cytoplasmic CD20 immunolabeling. IHC for CD20, hematoxylin counterstain, 400x. **i** Infiltrating cells are negative to CD3 immunolabeling, very few small mature, non-neoplastic lymphocytes show positive CD3 immunolabeling. IHC for CD3, hematoxylin counterstain, 400x

The 28 lymphoma and the leukemia cases were classified by applying the adapted WHO classification of lymphomas for animals [31] with the following results (Table 5 and Additional file 3). One (3.4%) out of the 29 lymphoid neoplasms was diagnosed as a precursor lymphoid cell neoplasm which was one B-lymphoblastic leukemia (B-LBL). Within the mature B-cell neoplasms, one (3.4%) out of 29 was classified as B-cell small lymphocytic lymphoma (B-SLL) and 16 (55.2%) out of 29 were diagnosed as Diffuse Large B-cell Lymphoma (DLBCL); eight (50%) out of these 16 were subclassified as centroblastic (DLBCL-CB). The remaining eight (50%) DLBCL could not be further classified and remained as unspecified DLBCL. Within the mature T-cell neoplasms, three (10.3%) out of 29 were classified as nodal T-cell lymphomas and subclassified as unspecified, peripheral T-cell lymphoma (PTCL) (Fig. 2). Five (17.2%) out of 29 were non-B non-T lymphomas (null cell lymphomas). Three (10.3%) out of the 29 lymphoid neoplasms could not be further characterized and remained as unclassified. Cases 19 and 46 presented morphological and immunohistochemical (B and T cell markers) features which did not fit into any of the current WHO categories described in domestic animals. For case 42 immunohistochemical characterization was not available.

**Table 5:** Lymphoid neoplasms detected in slaughtered pigs in Catalonia between January 1998 and April 2018

Classification		Cases
Precursor Lymphoid Cell Neoplasms		1
*• B-lymphoblastic leukemia/lymphoma*	*B-LBL*	*1*
*• T-lymphoblastic leukemia/lymphoma*	*T-LBL*	*0*
B-Cell Neoplasms		
Mature (Peripheral) B-Cell Neoplasms		17
B-cell chronic lymphocytic leukemia/small lymphocytic lymphoma	B-CLL/SLL	1
Diffuse large B-cell lymphoma	DLBCL	16
*• Centroblastic*	*DLBCL-CB*	*8*
*• Immunoblastic*	*DLBCL-IB*	*0*
*• T-cell rich B-cell lymphoma*	*TCRBCL*	*0*
*• Anaplastic large cell lymphoma*	*–*	*0*
*• Lymphomatoid granulomatosis*	*LYG*	*0*
*• None of the above*	*–*	*8*
Follicular-derived B-cell lymphomas	–	0
T-Cell and NK-Cell Neoplasms		
Mature (Peripheral) T Cell Neoplasms		3
Nodal T-cell lymphoma	–	2
*• T-zone lymphomas*	*TZL*	*0*
*• Anaplastic large T-cell lymphoma*	*ALTCL*	*0*
*• Angioimmunoblastic T-cell lymphoma*	*AITCL*	*0*
*• Unspecified, peripheral T-cell lymphoma*	*PTCL*	*2*
Enteropathy associated t-cell lymphoma	EATL	0
*• EATL type I (large cell neoplasms)*	*EATL-I*	*0*
*• EATL type II (small to intermediate cell neoplasms)*	*EATL-II*	*0*
Extranodal t-cell lymphoma	–	1
*• Hepatosplenic T-cell lymphoma*	*HS-TCL*	*0*
*• Hepatocytotropic T-cell lymphoma*	*HC-TCL*	*0*
*• Unspecified, peripheral T-cell lymphoma*	*PTCL*	*1*
Cutaneous T-cell lymphoma	CTCL	0
*• Epitheliotropic*	*–*	*0*
*• Non-epitheliotropic*	*–*	*0*
T-cell large granular lymphocytic leukemia	TC-LGL	0
*• Acute T-cell large granular lymphocytic leukemia*	*ATC-LGL*	*0*
*• Chronic T-cell large granular lymphocytic leukemia*	*CTC-LGL*	*0*
Non-B Non-T lymphomas^a^	*Null cell*	5
Unclassified^b^	–	3

The WHO lymphoma classification adapted to domestic animals was used [31, 74]. ^a^Lack of immunoreactivity for B or T cell lineages. ^b^ Neoplasms that do not classify in any one of the given categories based on cellular morphology and B-cell or T-cell immunoreactivity

## Discussion

An increase in the detection of neoplasms in pigs has been noticed in the last 10 years in Catalonia since the implementation of the SESC, a service that provides diagnostic support to meat inspectors. The highest incidence of detected cases was in 2012, that was less than one case for every 2 million pigs. However, the incidence of neoplastic cases in pigs over the total number of pigs slaughtered per year in the region is very low and thus represents a close to negligible economic impact (Additional file 4). The low number of cases submitted could be explained in part because enquiries sent to the SESC are voluntary for the VMI and, therefore, not all cases are submitted, particularly those easily macroscopically recognizable (e.g. lymphomas, melanomas). Therefore, in these cases, a macroscopic diagnosis is established without further laboratory confirmation. At slaughter, condemnation of a carcass is based on gross findings, on the regulations in place and on the inspector’s experience. Therefore, submitted cases are often those which are uncommon or rare and cannot be recognized easily. Learning motivation of the inspectors also influences whether a case is submitted or not. Consequently, the information obtained in this case series most likely underestimates the real incidence of neoplastic lesions in Catalonia during the studied period. These results agreed with previous publications in the veterinary literature, where a low incidence of neoplasms in this species has been reported [3, 17–26, 28], particularly due to the fact that pigs are usually slaughtered before reaching maturity, at the age of 5–6 months [1] and also due to certain underreporting as discussed above.

Despite the negligible economic impact of neoplastic lesions in swine, a scientific concern prevails in the surveillance of neoplastic diseases. Spontaneous carcinogenesis in the pig represents a useful model, given the anatomical and physiological similarities between pigs and humans, as well as the broad availability of this species [74, 75]. The availability of a neoplasm database in swine is required to better see emergence tendencies in this type of lesions and to notice any variations in the incidence (e.g.: outbreaks) that could take place.

In this study, neoplasms were observed more in fattening pigs and in regards of gender predisposition, it was higher in adult females compared to males. These biased data agree with the previous literature [76] and it is also explained by the porcine slaughter annual data from Catalonia where the vast majority of animals sent to slaughter are fattening pigs (> 90% of the total number of animals slaughtered per year) followed by sows (representing up to 2% of the total) (data from Ministry of Agriculture and Fisheries, Food and Environment (MAPAMA); Spain; years 2007 to 2018). For that reason, is not rare to observe more detected neoplasms in young pigs and in adult females, although proportionally, as expected, the incidence is superior in older animals.

In this case series, the most frequent neoplasm was lymphoma since 50% of the submitted cases fell in this category. Lymphoid neoplasms are the most common tumors in pigs and this is reflected in the larger number of lymphoid neoplasms compared to other types both in our study and in previously published literature [3–13]. In this study, 35.6% of the cases were adult females, which also agrees with previously described prevalence in this species [1, 3, 24–26, 28, 31, 77]. An approach to the human WHO lymphoma classification using the one as applied in animals allowed the identification of one precursor lymphoid cell neoplasm classified as B-LBL. Within the mature B-cell neoplasms, one B-SLL and 16 DLBCL including eight DLBCL-CB and eight unspecified DLBCL were identified. Within the mature T cell neoplasms, three PTCL were identified. Five null cell lymphomas were also reported, and three remained as unclassified. Only 5 of the described 15 lymphoma subtypes for animals were identified in this case series [31]. As listed above, in this study, lymphomas were predominantly B-cell in origin and classified as DLBCL based on morphology and immunophenotype and the most affected organs where liver, kidney, spleen and lymph nodes. The results are comparable to published data in dogs [31, 77, 78], where DLBCL is the most commonly diagnosed. On the other hand, these results differ from previous studies in swine, where DLBCL represented a small subset [11] and goats, where they are predominantly classified as T cell lymphomas [79]. The second most represented subtype in the present study was the null cell lymphoma based on morphological features and lack of CD3/CD20 antigen positivity. The classification of lymphoid neoplasms was based on the WHO guidelines, which includes characteristics of tissue architecture, cellular morphology and immunophenotyping. The major limitations of the present study were (1) availability of further immunohistochemical testing (only CD20 and CD3 markers were used) and (2) accuracy of reported clinical data. However, the present work is the first series of swine lymphoid neoplasms classification based on current WHO guidelines.

Other round cell neoplastic entities found were leukemia, mast cell tumor and a non-classified malignant round cell tumor. Mast cell tumors are uncommon in pigs and rarely reported. Only 2 cases of mast cell tumors [39, 58] were collected in this literature review, one of them [58] included in our case series. On the other hand, histiocytic diseases are rare in pigs. Congenital histiocytosis and a malignant fibrous histiocytoma have been reported in the literature [43, 66]. A case of a histiocytic sarcoma affecting liver, kidney and lymph nodes in a 2–3-year-old crossbreed female is reported in this case series.

Melanomas were also submitted. Melanocytomas and malignant melanomas have been reported in pigs [14–16, 24]. In 1990, Bundza and Feltmate detected a high rate of spontaneously regressing melanomas (174 out of 220 cases). Later on, an immunohistochemical study suggested a key role of the local cellular inflammatory response in the regression of cutaneous melanomas and melanocytomas in swine [16]. The reported high regression of this type of neoplasia could explain the low number of cases detected in our study-period. Moreover, melanomas are well-characterized and recognizable gross lesions and, thus, likely undersubmitted.

Nephroblastoma, an embryonal tumor, was the third neoplasm in frequency in the present series. The characteristic features of porcine nephroblastoma are well known and our observations correspond with those found in the literature [17, 24, 26, 32, 33]. Moreover, its true frequency is probably higher than the one reported here, since again this neoplasm is relatively easy to be recognized and, therefore, unlikely to be submitted for further characterization.

Endocrine cell neoplasia was represented by a malignant pheochromocytoma in a 2.5 year-old sow [63], a rarely observed neoplasm reported from a case submitted to the SESC-SDPV within the period considered by this retrospective study [18] .

Mesenchymal-spindle cell neoplasms were represented by fibropapilloma, osteosarcoma, osteochondromatosis, liposarcoma, hemangiosarcoma, and one undifferentiated sarcoma. The fibropapilloma in swine is thought to be of viral origin, caused by a *Sus scrofa* papillomavirus [80, 81]. Congenital cutaneous fibropapillomatosis with no detected virus infection has been reported in a piglet [59]. In the reported case in this study, no further tests were available to detect or rule out a viral origin. New neoplastic descriptions, not previously reported in pigs, have been published during this period from cases submitted to the SESC-SDPV, including one case of osteochondromatosis in a 2-year-old female domestic pig [64] and a case of liposarcoma in a 2.5-year-old, mixed breed commercial sow, detected during meat inspection [68]. Initial description of the lesions prompted the detection and correct diagnoses of further cases, included in this case series: a second case of osteochondromatosis and of liposarcoma.

Nervous system tumors were represented by PNST. In slaughtered pigs, five benign [24, 48, 72], and two malignant PNST [69, 71] have been reported. The animals affected by malignant PNST were adult (sow) and the organs included lung and thoracic limb. In this case series, a PNST affecting the kidney of a fattening male was diagnosed.

Epithelial cell tumors were uncommon during this period, including a hepatoma, a papillary cystadenocarcinoma (most likely of ovary origin) and one non-determined round/polygonal cell tumor (most likely mesothelioma). Tumors primarily arising from the liver have been previously reported in slaughter pigs [21, 24, 26, 47, 67] and in pot-bellied pigs, with a higher number of cases detected in this breed [30]. In this case series, a single case of benign hepatic tumor was diagnosed.

Neoplasms of the reproductive system in pigs are uncommon and the most commonly diagnosed are leiomyoma, fibroma, cystadenoma, fibroleiomyoma and carcinoma [24, 34, 40, 45, 46, 82]. Few ovarian epithelial neoplasms have been reported in pigs [23, 29]. This type of tumor arises from the surface of the coelomic epithelium and is very important in women but in domestic animals is only common in the bitch [83]. In this case series the papillary cystadenocarcinoma was the only neoplasm detected, primarily arising from the female reproductive system. Other neoplasms affecting organs of the reproductive system were only metastatic lymphomas, 2 cases in the uterus and 4 cases in the ovary.

Despite the advances in histopathology, molecular testing, and approaches to standardization of tumor classification in domestic animals, especially farm animals, classification of neoplastic lesions according to standard medical schemes still represents a challenge. This is mainly due to the lack of studies with adequate case number per group and further molecular and immunohistochemical characterization.

## Conlusions

In summary, a case series of 56 neoplasms detected in porcine slaughterhouses in Catalonia is presented. Twenty nine of them were lymphoid neoplasms that were further characterized and classified using the WHO classification adapted for animals [31]. The existence of SESC, a slaughterhouse support network, allowed the compilation of comprehensive data available for future epidemiological and pathological studies, particularly about less commonly reported lesions such as neoplasms in pigs.

## Materials and methods

### Literature review

The following online international databases were used: PubMed, Sage Journals and ScienceDirect for published data on the topic (Additional file 1). The following search wording was used: [“swine neoplasm” OR “pig neoplasm” OR “sow neoplasm”] and [“swine tumor” OR “pig tumor” OR “sow tumor”]. Specific search for each neoplasm detected in our case series was also conducted with the phrase: [(“swine” OR “pig” OR “sow” OR “boar”) AND (“type of tumor (e.g. lymphoma”)]. The databases were searched for papers published up to January 2021. Papers were excluded if any of the following criteria were met: (i) experimental study; (ii) Vietnamese, pot-bellied, miniature pigs; (iii) wild boar. Textbooks were not included in the search.

The retrieved papers were grouped as case reports (single case reports of different types of neoplasm), case series study (large number of cases studied and retrospective studies of one or more types of neoplasm) or survey (abattoir/meat inspection surveys).

### Data collection

Samples were obtained from the Slaughterhouse Support Network (*Servei de Suport a Escorxadors* [SESC] IRTA-CreSA]) and in collaboration with the Veterinary Pathology Diagnostic Service (*Servei de diagnòstic de Patologia Veterinària* [SDPV]) from the Autonomous University of Barcelona (*Universitat Autònoma de Barcelona* [UAB]). The SESC, established in 2008, is a slaughterhouse support network with the main goal of providing meat inspectors with continuing education to improve their ability to diagnose lesions they might come across in abattoirs of Catalonia [84]. All cases with a final confirmed diagnosis of neoplasia were selected from SDPV (1998–2007) and SESC (2008–2018) databases. Most of the submitted cases included samples for laboratory analysis and some cases (10–12%) consisted of telematic enquiries only. The latter have not been included in the study since their histopathological characterization was not possible.

Information of each enquiry from SESC was submitted through a web-app and included details of the origin of the sample, species, breed, age and sex of the animals, the organs involved, suspected conditions outlined by the veterinary meat inspectors (VMI) and macroscopic pictures of the lesions.

Fresh samples were delivered to SESC laboratory mostly within 24 h and were kept refrigerated. If not feasible, the recommendation was to send the sample half frozen and half fixed in formaldehyde 4% [84]. However, a few samples were submitted frozen, thus, hindering a fine characterization of the morphology due to freezing artifacts. For histopathological analysis, samples were fixed by immersion in 10% buffered formalin and embedded in paraffin for subsequent processing. Sections were stained with hematoxylin and eosin (HE). Immunohistochemistry (IHC) (CD3, CD20, vimentin, pancytokeratin, c-Kit, and S100) and special stains (Ziehl-Neelsen, Sudan Red, Toluidine blue and Congo red) were performed when required. The histopathological reports were written by ECVP-certified pathologists.

### Lymphoma classification in domestic animals

Lymphoid tumors in domestic animals are classified according to anatomic location and organs affected into: (1) multicentric, (2) thymic/mediastinal, (3) gastrointestinal, (4) cutaneous, (5) extranodal, and (6) central nervous system (CNS). In pigs, the most reported forms are (1) multicentric, and (2) thymic/mediastinal. The current classification of lymphoid neoplasms adopted by the WHO [31] was used in the present case series to further classify the reported lymphoid neoplasm cases.

The morphological features analyzed in each case were: (1) pattern, diffuse or follicular; (2) nuclear size; (3) cytoplasm size; (4) mitoses; and (5) phenotype (T cell or B cell). Nuclear size was determined as small (< 1.5x of a red blood cell (RBC) size), intermediate (1.5-2x RBC size) or large (>2x RBC size). For the mitotic index, figures were counted in 10 random (40x) high power fields (HPF). Low grade was given for lymphomas with 0 to 5 mitoses × 40/HPF, medium grade for 6 to 10 mitoses × 40/HPF and high grade for > 10 mitoses × 40/HPF. In diffuse large B-cell lymphoma (DLBCL), differentiation between *immunoblastic* (single central prominent nucleolus) and *centroblastic* (cells with multiple nucleoli, often located at the nuclear periphery) was made where possible. In cases where both types of nucleolar arrangement were present, *immunoblastic* was only assigned when at least 90% of nuclei were of that type [85].

The location of the lesions was extracted from the provided clinical history in the slaughterhouse submission forms.

Phenotype of lymphomas was established by means of immunohistochemical labelling for B and T cell using anti-CD20 and CD3 primary antibodies, respectively. Paraffin-embedded tissue sections (3–5 μm in thickness) were air dried and further dried at 60 °C overnight. Immunohistochemistry was performed using an Autostainer Plus (Dako, Agilent) machine. Prior to immunohistochemistry, and common for both protocols, sections were dewaxed and epitope retrieval was performed using Target retrieval solution Low pH (50X) (Dako, K8005) for 20 min at 98 °C using a PT Link (Dako, Agilent). Washings were performed using the EnVision flex wash buffer (20x) (Dako, K8000). Quenching of endogenous peroxidase was performed by 30 min of incubation with Peroxidase-Blocking Solution (Dako REAL, S2023).

For CD3, the primary antibody Rabbit polyclonal Anti-CD3 (Dako, A0452) at 1:100 dilution was incubated for 40 min at room temperature. For CD20, the primary antibody Rabbit polyclonal Anti-CD20 (Thermo Fisher Scientific, PA5-32313) at a dilution of 1:200 was incubated for 40 min at room temperature.

In both protocols, the secondary antibody used was the REAL Envision HRP Rabbit/Mouse (Dako, K5007) reagent. Antigen–antibody complexes were revealed with 3–3′-diaminobenzidine (Dako, K3468), with the same time exposure (5 min). Sections were counterstained with hematoxylin (Mayer, MHS1) and mounted with DPX Mounting Medium. Specificity of staining was confirmed by omission of the primary antibody.

Histology pictures were acquired with a Leica DM2500 microscope using the 3.1-megapixel digital microscope camera Leica EC3 in conjunction with the Leica Application Suite (LAS) EZ software.

## Supplementary Information

**Additional file 1 **Summary of the systematic literature review of the domestic pig (*Sus scrofa domesticus*) neoplasms reported between 1956 and 2021. Miniature breeds not included. *NA = Not Available; d = days; m = months; w = weeks; y = years; F=Female; M = Male.*

**Additional file 2 **SESC-SDPV cases summary including information about age, breed, sex, organ, gross findings, meat inspector suspicion and final diagnosis. *NA = Not Available; N/A = Not applicable; Piglet = 1.5 to 3 months; Fattening = 4 months to 1 year; Adult (sow) = Breeding female; Adult (boar) = Breeding male; F=Female; M = Male; (+) = positive; (−) = negative; DLBCL = Diffuse large B-cell Lymphoma, unspecified; DLBCL-CB=Diffuse large B-cell Lymphoma - Centroblastic subtype; B-SLL = B-cell Small lymphocytic lymphoma; PTCL = Peripheral T-cell Lymphomas, unspecified; Null cell = Non-T non B; B-LBL = B-lymphoblastic leukemia; UC=Unclassified.*

**Additional file 3 **SESC-SDPV Lymphoma cases and classification following the current classification of lymphoid neoplasms for domestic animals adopted by the WHO. *NA = Not Available; N/A = Not applicable; Piglet = 1.5 to 3 months; Fattening = 4 months to 1 year; Adult (sow) = Breeding female; Adult (boar) = Breeding male; F=Female; M = Male; LN = Lymph node; DLBCL = Diffuse large B-cell Lymphoma, unspecified; DLBCL-CB=Diffuse large B-cell Lymphoma - Centroblastic subtype; B-SLL = B-cell Small lymphocytic lymphoma; PTCL = Peripheral T-cell Lymphomas, unspecified; Null cell = Non-T non B; B-LBL = B-lymphoblastic leukemia; UC=Unclassified.*

**Additional file 4 ***Calculated percentage of neoplasia submitted from slaughtered pigs in Catalonia between January 2007 to April 2018* (data from Ministry of Agriculture and Fisheries, Food and Environment (MAPAMA), Spain; years 2007 to 2018). *N = number; NA = Not available data.*

## Data Availability

All data generated or analysed during this study are included in this published article [and its additional files(s)].

## Abbreviations

B-LBL: B-lymphoblastic leukemia
B-SLL: B-cell small lymphocytic lymphoma
CD3: Cluster of differentiation 3
CD20: Cluster of differentiation 20
c-Kit: Receptor tyrosine kinase
CNS: Central Nervous System
DLBCL: Diffuse Large B-cell Lymphoma
DLBCL-CB: Centroblastic Diffuse Large B-cell Lymphoma
DPX: Dibutylphthalate Polystyrene Xylene
ECVP: European College of Veterinary Pathologists
HE: Hematoxylin and eosin
HPF: High Power Fields
HRP: Horseradish peroxidase
IHC: Immunohistochemistry
MAPAMA: Ministry of Agriculture and Fisheries, Food and Environment
null cell lymphomas: Non-B non-T lymphomas
PNST: Peripheral nerve sheath tumour
PTCL: Peripheral T-cell lymphoma
RBC: Red Blood Cell
SCC: Squamous cell carcinoma
SDPV: “*Servei de diagnòtic de Patologia Veterinària”*
SESC: “*Servei de Suport a Escorxadors”*
VMI: Veterinary meat inspector
WHO: World Health Organization
