# Supplementary material for: Neoplastic lesions in domestic pigs detected at slaughter: literature review and a 20-year review (1998–2018) of carcass inspection in Catalonia
Source: Porcine Health Manag. 2021 Apr 7;7:30. doi: 10.1186/s40813-021-00207-0 (PMC8025367; doi:10.1186/s40813-021-00207-0)
Supplement: Supplementary file 4 — Additional file 4 Calculated percentage of neoplasia submitted from slaughtered pigs in Catalonia between January 2007 to April 2018 (data from Ministry of Agriculture and Fisheries, Food and Environment (MAPAMA), Spain; years 2007 to 2018). N = number; NA = Not available data. [file 40813_2021_207_MOESM4_ESM.docx]

| Year | Nº detected cases | Nº slaughtered pigs | Proportion (x 1.000.000) |
| --- | --- | --- | --- |
| 2007 | 0 | 16.353.138 | 0,000 |
| 2008 | 1 | 16.729.435 | 0,060 |
| 2009 | 6 | 16.717.935 | 0,299 |
| 2010 | 2 | 16.898.418 | 0,118 |
| 2011 | 6 | 17.449.951 | 0,344 |
| 2012 | 8 | 18.042.794 | 0,443 |
| 2013 | 5 | 18.593.752 | 0,269 |
| 2014 | 8 | 19.362.768 | 0,362 |
| 2015 | 4 | 19.854.375 | 0,201 |
| 2016 | 5 | 21.684.315 | 0,231 |
| 2017 | 5 | 21.646.356 | 0,023 |
| 2018 | 2 | 22.430.128 | 0,009 |
